# Fever and Heat Centers

**Published:** 1890-06

**Authors:** 


### Selections.

Mosso (Hugo). "The laws of fever in relation to the cerebral heat centers."—*Archives of Experimental Pathology*, Vol. XXVI.

Gottlieb (R). "Experimental researches on the mode of action of drugs that lower temperature."—*Archives of Experimental Pathology*, Vol. XXVI., p. 419—453.

Hale White (W.). "Brain lesions and heat."—*Journal of Physiology*, Vol. XI., Jan., 1890.

It is said with some truth that to medical practitioners of the present day the clinical thermometer and the patient's temperature are more than to the practitioners of the past generation were the tactus doctus and the radial pulse. The thermometer when carefully used will give accurate information as to bodily temperature ; but when that information has been gained, what further insight does it allow into the body's natural or morbid processes ? The temperature of the animal body is the resultant of two great reciprocal processes within it going forward side by side. On the one hand, there is production in the tissues ; on the other, loss at the surface. Bodily warmth is, as it were, balance in hand, where thermogenesis is debtor and thermolysis creditor. With the thermometer can at best only be shown the degree of this excess ; and to know it is often of inestimable value. Yet how much more satisfactory were it only possible for the practitioner to say, "This patient's temperature is high because the amount of heat the tissues are producing is greater than in health"; or, again, "This patient's temperature is too high because the heat arising within does not escape from the body so freely as it should."

Concerning the manner in which heat is lost to the body, much more is clear than is the case with the manner of the thermogenesis. Nearly nine-tenths of the dissipation of heat occurs at the surface of the skin. As to the mechanism of this escape by the skin, and the nicety of the regulation of it, our knowledge is comparatively abundant. The body temperature, apart from a diurnal rhythm of variation, is kept practically constant and independent of climate, weather and changes in food or activity. The amount of blood allowed to stream through the cool surface region, as also the cooling power of every unit of that surface, is capable of variation, and is continually adjusted to circumstances, its control being in the grasp of the central nervous system. But of our knowledge of the thermogenetic processes of the body, what may be said? How and to what extent does it remain constant? What is its site, and in what way is it capable of being controlled? To such questions as these many have attempted to obtain reply, and, at the present day, results of experiment seem to make an answer, in some sort, possible. The heat of the body derived from the freeing of energy latent in the chemical substances of the tissues, is the result of processes, which are, broadly speaking, processes of oxidation. Every living particle of the body produces heat as certainly as it, in virtue of living, displays katabolic activity, as opposed to assimilative or anabolic activity. But some tissues are larger producers of heat than are others, as in arum the flower-bud produces more heat than does the stalk. The blood leaving the liver is the warmest in the body, and the liver, holding a quarter of the total blood, is well considered a main furnace in the body. Still more important must be the heat-production by voluntary muscles. It is not merely that at every repetition of that extreme form of its activity which constitutes "contraction" an outburst of heat takes place within the muscle. The continuous gentler action of the muscle which, viewed from one aspect, is manifest as its "tenacity," is probably the great thermogenetic agent of the animal organism. For this tonus of the voluntary muscles their connection with the central nervous system is essential. That nexus ruptured tone is lost, and the toneless muscles no longer take their wonted share in thermogenesis, other things remaining equal, the production of heat and the body's temperature diminish in consequence.

It was Brodie, eighty years ago, who first showed by direct observation that the amount of heat production is under the control of the central nervous system. It was not until nearly fifty years later that his observations acquired additional interest from the experiments of Tscheschichin in Du Bois Reymond's laboratory. Tscheschichin remarked on the rabbit, that if the pons were divided completely across, the body temperature rose more than 3° C. Besides the increase of temperature there was increase of reflex irritability, and not infrequently general convulsions set in. Four years later, Heidenhain published experiments by two of his pupils, showing that a simple puncture of the brain is followed by increase of temperature with more certainty than were the lesions carried out in Du Bois' laboratory. Also that puncture with two needles is more effectual than puncture with one; in fact, that the result is due to the traumatic irritation of the part injured. Heidenhain's experiments were interrupted by the outbreak of the Franco-German War, and it was not until two years after the war that a paper again appeared in Pflüger's Archives, dealing with the matter. In this Schreiber recorded that there was no rise of temperature, on the contrary a fall, unless the rabbit were shielded from loss of warmth, by being wrapped in non-conducting material. This seemed to point to the previously recorded high temperatures as merely affairs of surface, suggesting that in some way the injury to the brain caused flushing of the surface with blood, presumably through vaso-paralysis of the skin. But it was the temperature in the rectum that had been measured in Heidenhain's laboratory. Six years later Wood published the first calorimetrical observations on the subject, conducted on dogs, not on rabbits. The calorimeter, as compared with the thermometer, has the advantage of giving the actual amounts of heat lost from the body and of heat produced by the body for the whole time, which may be prolonged to many hours, over which the observation with the instrument is extended. After the spinal cord had been cut across in the upper thoracic region, *i. e.*, above the spinal origin of the splanchnic nerves, Wood observed a diminution in the amount of heat given off from the body, accompanied by an increased production of heat in the body. If the division of the cord were quite high up, near the bulb, there was, on the other hand, more heat given off, and, as before, the greater production of heat within the body. He concluded that this was due to the cutting off of a cerebral influence that restrains the chemical activity of the tissues that produce heat. In this connection it is memorable that Eulenberg and Landois had previously shown that destruction of the motor cortex causes a heightened temperature of the paralyzed limb, referable to vaso-motor paralysis of the skin of the paralyzed part. The researches of Wood were published in 1880. Since that time a considerable number of investigators have entered the field. Ott (1884), Richet (1884), Aronsohn and Sachs (1885), Girard (1887), all concur in localizing in the brain near the corpus striatum "centres," injury to which brings about an increase of the body's temperature. The experiments of the two latter observers, which were very numerous and careful, show that both mechanical and electrical stimulation of the region in question can raise the temperature of the rabbit by as many as 4° C. A corresponding increase in the amount of carbonic acid gas and of urea given off by the animal they found to occur coincidently with the rise in temperature. Ott, using the calorimeter, localized three centres in the corpus striatum, injury to any one of which causes increased thermogenesis ; he also believes a fourth centre to exist in the anterior part of the optic thalamus. Recently he has contributed to *Brain* clinical evidence in favor of his experimental localizations.

It appears, therefore, undeniable, that injuries of quite small extent in certain regions of the brain do produce a fever temperature, and that the heightened temperature is referable at least in part to increased thermogenesis. The obscurity that surrounds the matter attaches chiefly to the uncertainty with which the fever temperature is found to occur, and to the fact that it may follow or it may not, in experiments which to all appearance have been similar. Very welcome, therefore, are three most recent contributions to our knowledge of the subject.

Dr. Hugo Mosso has conducted a series of experiments in the laboratory of his brother, the distinguished physiologist in Turin. The dog was chosen as the subject of his research, on account of the greater constancy of the body temperature in the dog than in the rabbit. The animal's temperature was measured by a thermometer in the rectum. Carefully controled observations were made determining the variations of the rectal temperature which occur normally in the dog. Besides thermometry Mosso employed also calorimetry, using a very ingenious air calorimeter, which he devised especially for his experiments. The instrument was so arranged as to permit great facility of manipulation. From very numerous experiments conducted along both lines of method, he concludes that small lesions neither of the cerebral cortex nor of the basal ganglia produce in the dog any rise of temperature or increased heat production comparable to the effects observed in rabbits. He believes, however, that psychical excitement, such for instance as that occasioned by the binding of the animal down, may increase the temperature very considerably, and he has also found that occasionally large lesions of the basal portions of the brain do cause very marked increase of the body temperature and of thermogenesis. A particularly interesting portion of Dr. Mosso's paper is that in which he deals with the relation of cerebral action to ordinary fever. Mosso was the first to show that hypodermic injection of cocain produces a fever temperature, and his observation has been recently confirmed by Professor Reichert. He finds that in this fever injury of the cerebral hemisphere has no obvious effect upon the increased heat production or the heightened temperature, nor does it upon the temperature or thermogenesis of dogs in which a septic fever has been induced.

A second recent paper is by Gottlieb, and comes to us from Professor Schmiedeberg's laboratory in Strasburg. In 1887, after Girard's paper in the *Archives* then directed by Vulpian, a practical issue of the question previously neglected, giving fresh interest to the whole discussion, was recognized. If there are heat centres in the brain, may not fever in some cases be due simply to disturbance of such centres ? If so, may the antipyretic drugs act upon fever through the cerebral heat centres? Gottlieb's object has been to ascertain the effect of antipyretic drugs upon the fever temperature produced by pricking the brain. His experiments were carried out upon rabbits and by thermometry, the temperature being taken in the rectum. He describes that wounding the brain with a rod an eighth of an inch in diameter causes unfailingly an immediate rise of the rectal temperature, amounting usually to four degrees centigrade, rapid in the first four hours and maintained for from eighteen to twenty-four hours. Antipyrin, quinine, or morphia in small doses, brings down this puncture temperature. He believes the drug produces its effect by acting upon the part of the cerebrum which has been irritated by the puncture. This it probably does, Gottlieb thinks, by diminishing (inhibiting) the thermogenesis of the thermogenic tissues through these cerebral centres. His argument is based on the somewhat *a priori* grounds that in this respect, as in others, morphia is not a poison for protoplasm in general, but for nervous centers, *i. e.*, protoplasm differentiated in a very special direction.

A third paper is by Hale White in the first number of the new volume of the *Journal of Physiology*. It is preliminary to more extended observations, in which the calorimeter, as well as the thermometer, is to be employed, but the carefulness evident in the present communication makes it a valuable contribution in itself. Dr. Hale White has at the outset attempted to gauge certain errors of observation that might arise from causes accidental to his experiment. His first care has been to determine the normal temperature of the animal on which his research depends, namely, the rabbit. He finds the temperature as constant as the observations of Palmer would have it to be. Palmer, as did Aronsohn and Sachs, found the temperature of the rabbit to oscillate in health between 38·8° C. and 39·8° C. only; Manassein and also Gottlieb found a similar range of variation of one degree cent. In the rabbits examined by Dr. Hale White, the temperature was found to oscillate between 101° Fah. and 103° Fah.

The effect of the administration of ether had also to be controlled; this was done with the result that the etherization was found only occasionally to produce an abnormal temperature, namely, an increase of 1.2° Fahrenheit. In Mosso's experiments, presumably in Gottlieb's also, no anæsthetic was administered. A further control needed was laying bare the dura mater and the puncturing of that membrane; in six experiments this was found on one occasion to cause a rise of temperature of 1.6° F. in the rectum. The slight injury to the grey matter of the cortex, caused by the passage of the sheathed puncturing needle through it, may also, it was found, be left out of consideration. Fourteen experiments made upon the white centrum ovale occasioned no notable rise of temperature, except in four cases, in which the grey matter of the corpus striatum was found to have been also slightly damaged.

Of twenty-two experiments in which the corpus striatum was the part principally injured—discounting one in which very evident "shock" was observed to follow to puncture—eighteen gave an increase of the rectal temperature to 1° F. above normal, and seven of these an increase to 3° F. above normal. This amount of rise agrees very fairly with that obtained by Gottlieb after his punctures, and the duration and course of the fever appear also to agree with Gottlieb's charts. From eight experiments in which the optic thalamus was the part most injured, again omitting one in which the severity of the lesion produced obvious shock, it would appear that the increase of rectal temperature is not so great as after lesions of the corpus striatum; but is sufficiently marked as compared with results from parts other than the striata. As Dr. Hale White remarks, the experiments indicate that the striata and not the thalami possess in rabbits the power of modifying the body temperature, and that the surrounding white matter of the hemisphere possesses little or no such property.

Returning for a moment to the paper by Gottlieb, it is very interesting to note that the high temperature in this puncture febricula, sharp and brief lasting as it is, is, Gottlieb finds, brought down sometimes to a subnormal level by doses of morphia insufficient to bring about narcosis. If, as Gottlieb believes, the drug allays the fever heat, and also the fever, by quieting the centres that have been irritated by the lesion, it may be recalled that Heidenhain from the first concluded against Tscheschichin's original view of paralyzed inhibitory centres in the brain, and for the effect being due to stimulation and irritation by the trauma. This seems to be a most important contribution to the knowledge of fever. Fever may well be compounded of phenomena that are manifold in kind and cause—but if among them exists a class in which the increased temperature is mainly cerebral in origin, it would appear that there are certain drugs which can quiet and stay the tissue wear and tear that has been initiated by higher portions of the central nervous system. How far all such fever phenomena are independent of changes primarily of vaso-motor character is an interesting question, and one that seems to deserve more attention, and promise better repayment for attention, than might perhaps be gathered from most of the literature on the subject.—*Medical Chronicle, May, 1890.*

